# Spatial Analysis of *Astragalus verus* Olivier Dieback: Understanding Patterns and Implications for Conservation in Iranian Rangelands

**DOI:** 10.1002/ece3.73273

**Published:** 2026-04-08

**Authors:** Asieh Sheikhzadeh, Hossein Bashari, Mostafa Tarkesh Esfahani, Hong Hai Nguyen

**Affiliations:** ^1^ Department of Natural Resources Isfahan University of Technology Isfahan Iran; ^2^ Department of Forest Inventory and Planning, Faculty of Silviculture Vietnam National University of Forestry Hanoi Vietnam

**Keywords:** gradient analysis, plant species decline, rangeland, soil characteristics, species dieback

## Abstract

*Astragalus verus* Olivier, a species native to Iran, is experiencing dieback, particularly in the Zagros and Central Iran regions. This study examines whether dieback patterns are spatially non‐random and influenced by soil conditions. Twelve rangeland sites dominated by *A. verus* and showing signs of dieback were selected. At each site, a 100 m^2^ plot was randomly established to record the coordinates of all perennial species, focusing on live and dead *A. verus* individuals, followed by soil physicochemical analyses. Spatial patterns were evaluated using Ripley's K functions and nearest neighbor indices. Dieback showed positive correlations with sand content and electrical conductivity, and negative correlations with soil moisture, silt, and clay. While the overall plant community shifted from clumped to uniform spatial patterns with increasing spatial scale (distance from reference plants), the spatial distributions of live and dead *A. verus* individuals remained largely random, suggesting ecological independence. These findings indicated that *A. verus* mortality is likely driven by localized environmental stressors rather than density‐dependent interactions and highlight the value of spatial point pattern analysis for diagnosing species decline and guiding conservation in rangeland ecosystems.

## Graphical abstract

Astragalus verus is undergoing dieback in Iran's Zagros and Central regions, raising concerns about its conservation. This study applied spatial point pattern analysis to assess the relationship between plant mortality and soil properties across twelve rangeland sites. Results suggest that localized environmental stressors, rather than density‐dependent factors, are the primary drivers of dieback.

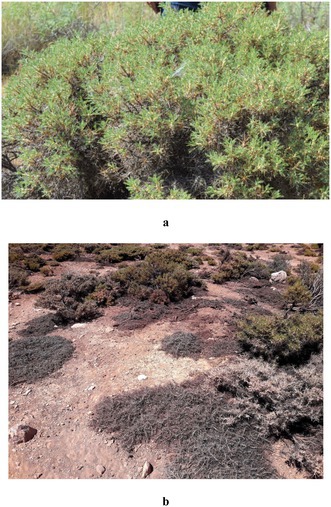

## 1 Introduction

Spatial patterns refer to the arrangement and distribution of organisms or features within a given area, such as a landscape or ecosystem. These patterns are fundamental aspects of ecology, offering insights into the organization and dynamics of ecosystems (Wiegand and Moloney 2004; Perry et al. 2008; Li and Reynolds 2023; Morera et al. 2025). In plant ecology, understanding spatial patterns is essential for interpreting ecological processes, assessing biodiversity, and informing conservation strategies (Maltez‐Mouro et al. 2007; Nizamani et al. 2023). Spatial patterns serve as key indicators of species interactions, interspecific relationships, and environmental influences on plant communities (Tsitsoni et al. 2003; Trifković and Yamamoto 2008). Clumped patterns often result from natural disturbances, reproductive strategies, or environmental heterogeneity, providing insight into habitat suitability and resource availability (Tsitsoni et al. 2003; Wiegand et al. 2007; Ramón et al. 2018). In contrast, regular or random patterns may reflect competitive interactions, habitat homogeneity, or past ecosystem disturbances (Trifković and Yamamoto 2008; Perry et al. 2008; de Bello et al. 2013; Scaggs et al. 2025).

Understanding the spatial dynamics of plant populations, particularly in the context of species decline, is crucial for effective conservation and rangeland management. Knowledge of spatial distribution aids in identifying vulnerable populations, implementing targeted conservation actions, and monitoring ecological responses to environmental stressors (Battisti et al. 2016; Yu et al. 2024). Patterns of dieback can reveal spatial signatures of ecological stress, helping to pinpoint contributing factors. Furthermore, identifying whether dieback follows predictable patterns enables proactive management strategies that can prevent further spread and promote population recovery. In this regard, advanced spatial analysis techniques offer powerful tools for accurately assessing species distributions and identifying areas in need of intervention.

Analytical methods such as Ripley's K function and the Nearest Neighbor Index allow researchers to quantify spatial patterns, detect clustering or dispersion, and explore the ecological drivers underlying these patterns (Ripley 1977; Stamatellos and Panourgias 2005; Chen and Shen 2024; Demichele et al. 2025). When integrated with environmental data, these techniques reveal relationships between spatial arrangement and abiotic stressors. This is particularly valuable for declining species facing threats from climate change, land‐use pressure, and soil degradation. One such species is *Astragalus verus* Olivier, a key component of Iran's rangeland ecosystems whose conservation has become increasingly urgent.

Iran hosts over 800 species of *Astragalus*, accounting for nearly 60% of the global diversity of the genus. These species cover approximately 17 million hectares of rangeland, making up about 19% of the country's total rangeland area (Masoumi 2006). Among them, *Astragalus verus* Olivier is native to mountainous regions of the Zagros and central Iran. Recognizable by its cushion‐ or hump‐shaped growth form, *A. verus* plays a vital role in soil stabilization by reducing erosion on slopes and enhancing moisture retention through its extensive root system. In addition to its ecological significance, the species produces tragacanth, a natural gum widely used in pharmaceuticals, cosmetics, and various industries. Despite its ecological and economic value, recent reports indicate a notable decline in *A. verus* populations, particularly in the Zagros and central regions (Safaei et al. 2018; Khodagholi and Saboohi 2019).

Given the importance of spatial patterns in understanding plant population dynamics and the growing concern over *A. verus* decline, a detailed spatial assessment of its dieback is essential. Studies on other plant species have shown that non‐random spatial patterns often emerge due to ecological pressures such as resource competition, soil degradation, and climatic variability (Maltez‐Mouro et al. 2007; Battisti et al. 2016). Since *A. verus* populations occur along altitudinal and edaphic gradients, including variations in elevation, slope position, soil texture, and moisture availability, and are subject to both anthropogenic and natural stressors, it is plausible that dieback patterns reflect these underlying environmental conditions. Identifying whether dieback areas show signs of clustering or dispersion can offer crucial insights into such ecological drivers.

The primary objective of this study is to investigate the spatial distribution and interactions between live and dead *A. verus* individuals in areas experiencing dieback. Using Ripley's K function and the Nearest Neighbor method, we assess whether the observed spatial distribution deviates from randomness and infer possible ecological mechanisms underlying dieback.

We hypothesize that the dieback of *A. verus* is spatially non‐random and is associated with localized soil degradation, particularly in areas with poor moisture retention and altered texture. This pattern would indicate ecological stress caused by hydraulic failure and edaphic filtering. This approach not only enhances conservation and restoration strategies for *A. verus* but also serves as a model for identifying the spatial distribution of other species of conservation concern. Moreover, the spatial analysis methods used in this study have broader applications in fields such as weed ecology, where understanding spatial spread across landscapes is equally critical.

## 2 Materials and Methods

### 2.1 Study Area

**FIGURE 1:**
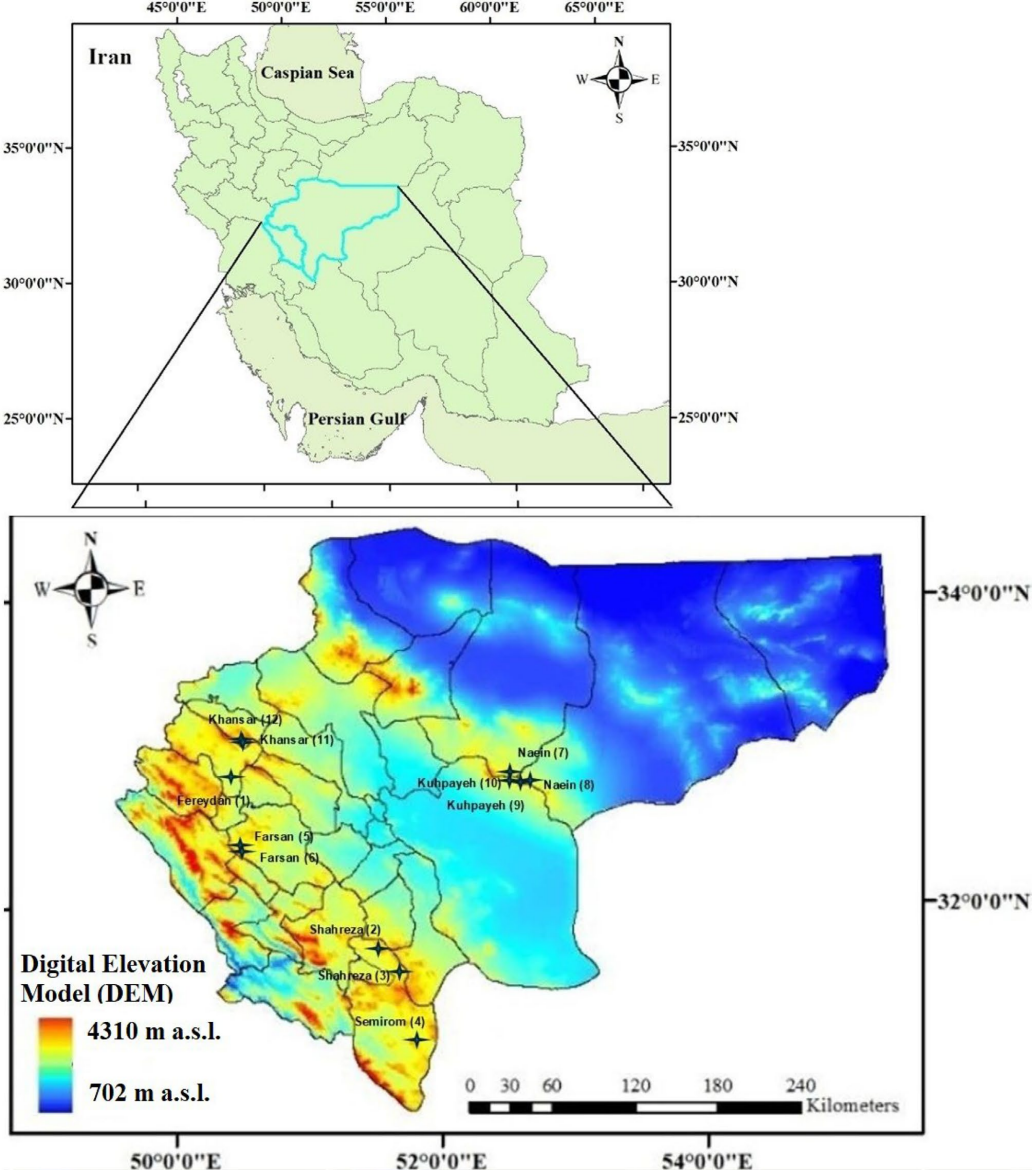
Location of the study area in Iran (top) and the distribution of sampling sites over the digital elevation model (DEM) map (bottom).

The study area encompasses portions of the Zagros and Central Iran regions, spanning approximately 123,027 km^2^ between latitudes 30°42′ and 34°30′ N, and longitudes 49°30′ and 55°00′ E (Figure 1). This area represents a significant habitat for *A. verus* in Iran, which has been experiencing widespread dieback of this species. According to the Emberger climate classification, the region is predominantly characterized by cold arid and cold semi‐arid climates. *A. verus* is primarily found on mountain slopes and hills within the physiographical units of the region.

The soil in the study area belongs to the Typic Haplocalcids subgroup, ranging from unevolved to evolved and from shallow to deep soils with varying textures from light to heavy (Vahabi 2005). The unique climate and physiographical characteristics of the region contribute to its significance for biodiversity, housing a diverse flora and fauna.

### 2.2 Morphological Diagnosis of *Astragalus verus*

The specimens of *Astragalus verus* used in this study were collected from field sites in Isfahan Province, Iran, and prepared as standard herbarium vouchers. Species identification was performed using the diagnostic keys provided in *Flora Iranica*, Papilionaceae VII: *Astragalus* V (Podlech et al. 2012). The collected specimens were compared with a verified herbarium voucher of *A. verus* (voucher number 13469, identified by Maassoumi) housed at the Herbarium of Isfahan University of Technology (international code: HIUT). Identification was carried out by Mrs. Bayat, an experienced herbarium specialist, following standard morphological procedures. In addition, species identity was confirmed through comparison with descriptions and photographic plates provided in *Flora Iranica*, Papilionaceae V: *Astragalus* III (Zarre et al. 2008), ensuring accurate taxonomic identification prior to subsequent ecological and spatial analyses.

**FIGURE 2:**
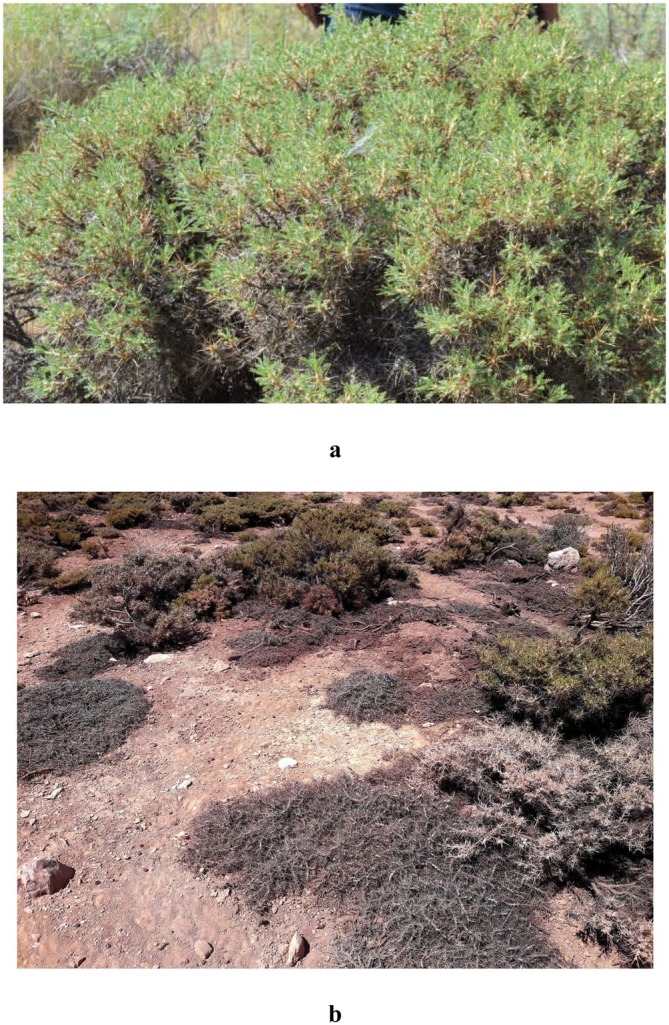
(a) A living *Astragalus verus* Olivier plant and (b) a dead *A. verus* plant observed in the rangelands of Isfahan Province, Iran.

*Astragalus verus* Olivier (Fabaceae: Papilionoideae, section *Rhacophorus*) is a perennial species with a cushion‐forming or spiny habit, ranging from 10 to 100 cm in height, occasionally shrubby, with a canopy diameter of 20–70 cm (Figure 2). Stems are intricately branched and entangled. The plant is gray to silvery, densely covered with white tomentose hairs, although some individuals may be sparsely pubescent or glabrous. Stipules are 5–10 mm long, creamy to whitish, adnate to at least the lower half of the petiole, densely floccose, and becoming glabrescent late with age. Leaves are 2–7 cm long, with 5–9 pairs of linear to elliptic‐oblong leaflets, 5–12 mm long, green to silvery. Inflorescences are spherical, oval, or cylindrical, with numerous flowers. The corolla is yellow or blue, occasionally purple; the standard petal (vexillum) is 6–10 mm long and distinctly constricted in the upper part. Wings and keel are 8–9 mm long, and the ovary is densely villous.

This species is morphologically similar to *A. microcephalus* Willd., but can be reliably distinguished by its creamy to whitish stipules, adnate to at least the lower half of the petiole, densely floccose, and becoming glabrescent late. In contrast, *A. microcephalus* has yellow stipules, adnate only to the middle of the petiole, sparsely floccose, and becoming glabrescent early (Podlech et al. 2012; Zarre et al. 2008).

### 2.3 Collecting Data

Twelve rangeland sites were selected based on three criteria: (i) dominance of *A. verus* within the plant community, (ii) presence of substantial dieback, defined as > 30% of individuals being completely dried or dead, and (iii) representation of different elevation strata (2300–2700 m). Because no formal mortality threshold has been established for *A. verus* , the > 30% cutoff was adopted as an ecologically meaningful indicator of significant dieback. Thresholds within the 20%–40% range are commonly used in dieback studies to distinguish background mortality from functionally important population reductions (Fensham et al. 2009). This threshold ensured the inclusion of sites with moderate to severe dieback and allowed consistent comparability among plots.

Sites with minimal utilization history were deliberately chosen to exclude the impact of improper harvesting of tragacanth gum, a known factor in *A. verus* decline (Azadrooh et al. 2020). This ensured that observed dieback patterns reflected natural ecological or environmental processes rather than anthropogenic influences.

To study the spatial distribution and dieback patterns of *A. verus* , species occurrence data were collected using GPS coordinates. Each site was represented by a 100 m^2^ plot (10 × 10 m), and sampling sites were spaced at least 20 km apart. Within each plot, the coordinates of all perennial species, including live and dead *A. verus* individuals, were recorded, with a hypothetical origin assigned as the reference point for spatial measurements.

The selected sites were grouped into three categories: western, southern, and eastern, to reflect major physiographic and climatic subregions. Grouping minimized within‐region variability and allowed for more ecologically meaningful comparisons. Group 1 (Sites 1, 5, 6, 11, 12) represents the western region; Group 2 (Sites 2, 3, 4) represents the southern region; and Group 3 (Sites 7–10) represents the eastern region (Figure 1, Table 1).

**TABLE 1:** Studied rangeland sites and environmental characteristics used to assess *Astragalus verus* Olivier dieback in Iranian rangelands.

Site number	Name of site	Elevation (m)	Annual Mean Temperature (°C)	Soil texture	Aspect	Dominant plant species	Total canopy Cover (%)	*Astragalus verus* dieback (%)
1	Fereidan	2415	11.7	Sandy Clay Loam	West	*Astragalus gossypinus*—*Phlomis olivieri*— *Astragalus verus*	15.1	30.7
2	Shahreza	2691	15.1	Clay Loam	Northeast	*Astragalus verus*	27.7	48.2
3	Shahreza	2642	15.1	Silty Loam	Southeast	*Echinops* sp. – *Scariola orientalis*— *Astragalus verus*	23.5	47.5
4	Semirom	2631	12.9	Sandy Clay Loam	Northeast	*Artemisia aucheri* – *Astragalus verus*	28.8	56
5	Farsan	2297	12.9	Clay	Northwest	*Astragalus verus*	33.5	28.2
6	Farsan	2352	12.9	Clay Loam	East	*Astragalus verus*	24.9	35.5
7	Naein	2474	17.4	Clay Loam	Northeast	*Artemisia aucheri* – *Astragalus verus*	20.4	89.3
8	Naein	2366	17.4	Clay Loam	South	*Artemisia aucheri* – *Astragalus verus*	23.5	69.0
9	Kuhpayeh	2364	15.3	Sandy Loam	South	*Stipa barbata* – *Acanthophyllum* sp.— *Astragalus verus*	16.0	75.0
10	Kuhpayeh	2510	15.3	Clay Loam	North	*Artemisia aucheri* – *Astragalus verus*	32.9	73.9
11	Khansar	2330	12.5	Clay	West	*Astragalus verus* – *Cousinia bakhtiarica*— *Centaurea virgata*	24.0	35.4
12	Khansar	2557	12.5	Clay	South	*Astragalus verus* – *Centaurea virgata* —*Cousinia bakhtiarica*	24.7	80

Soil samples were collected at depths of 0–25 cm and 25–50 cm from the center of each plot. These samples were analyzed for moisture content, soil texture (percentages of silt, clay, and sand), pH, and electrical conductivity to examine potential environmental gradients influencing dieback patterns (Carter and Gregorich 2006).

### 2.4 Data Analysis

#### 2.4.1 Diversity of Plant Community

Species diversity indices, including species dominance, Simpson's diversity, Shannon's diversity, and Chao's diversity, were calculated following Magurran (2021). All indices were computed using PAST 4 (Paleontological Statistics) software (https://www.nhm.uio.no/english/research/resources/past/).

Differences among the three site groups in species diversity indices were assessed using the Kruskal‐Wallis test. Normality and homogeneity of variances were examined, and because the data did not meet normality assumptions, a non‐parametric test was applied. Pairwise comparisons were conducted using the Mann–Whitney test. All statistical analyses were performed in IBM SPSS version 20.

To permit non‐parametric comparisons among groups, diversity indices were calculated separately for each site. Because the Kruskal–Wallis test evaluates differences based on the distribution of site‐level values, group means and measures of dispersion (e.g., SD or SE) were not computed. Accordingly, each index is reported at the site level in the Results.

#### 2.4.2 Soil Principal Component Analysis

Soil samples were collected from the center of each plot at depths of 0–25 cm and 25–50 cm for gradient analysis. Measurements included soil moisture content, texture (silt, clay, and sand percentages), pH, and electrical conductivity. To ensure a representative assessment of soil conditions, weighted mean values of soil properties from both depths were used in the analysis.

Ordination methods in the CANOCO 4.5 software package were applied to examine the relationship between plant factors and environmental variables. A Detrended Correspondence Analysis (DCA) was first conducted to determine the gradient length, which was found to be 0.816. As this indicated a relatively short gradient, Principal Component Analysis (PCA) was selected as the appropriate ordination method for analyzing linear relationships (Lepš 2014). PCA not only assesses variations in plant species composition and abundance in response to environmental variables but also identifies key soil properties influencing *A. verus* dieback.

The PCA‐derived Tri‐Plot diagram illustrates environmental variables and *A. verus* dieback percentage (calculated based on relative cover) using vectors. The direction of each vector represents the direction of maximum variation, while its length signifies the magnitude of the effect. In this ordination, environmental variables with longer vectors exhibit stronger correlations with plant factors, indicating a greater influence on species distribution and dieback patterns (Jangman et al. 1987).

#### 2.4.3 Nearest Neighbor Analysis

We employed Nearest Neighbor Analysis (NNA) to examine the spatial structure of *A. verus* populations. This method provides a detailed understanding of individual‐level interactions within plant communities, making it particularly useful for assessing fine‐scale spatial heterogeneity in rangeland ecosystems. Unlike broader spatial aggregation methods, NNA focuses on localized patterns by analyzing spatial relationships between individual plants and their nearest neighbors.

**FIGURE A1:**
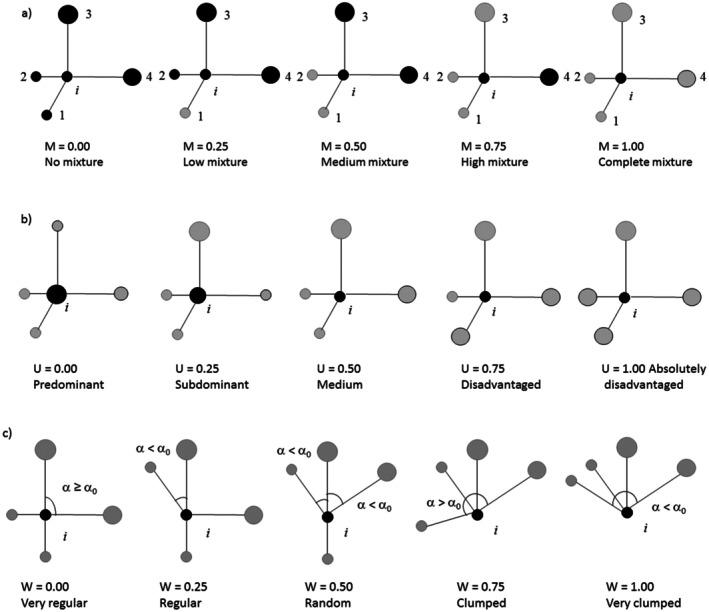
Definition of the spatial parameters: Mingling (a), Dominance (b) and Uniform Angle Index (c).

In rangeland ecosystems, plant stands consist of structural units, each forming a neighborhood with multiple species. To assess species spatial structure, we applied three structural indices proposed by Von Gadow and Hui (2002): species mingling (*M*), dominance (*U*), and uniform angle index (*W*). These indices characterize species composition, size variation, and spatial arrangement, respectively (Aguirre et al. 2003; Pommerening and Stoyan 2008; Hui et al. 2011; Padilla‐Martínez et al. 2024). Species mingling (*M*) quantifies species composition and spatial distribution by determining the proportion of the *n* nearest neighbors that belong to a different species than the reference plant (Figure A1 in Appendix A).

(1) $$M_{i}=\frac{1}{4}\sum_{j=1}^{4}v_{j}$$

*v*_*j*_ equals 1 if neighbor j belongs to a different species than reference species *i*; otherwise, *v*_*j*_ equals 0. Dominance (U) characterizes the variation in size between a reference species and its four closest neighbors. It is defined as the proportion of the n nearest neighbors that are smaller than the reference species (Figure A1 in Appendix A).

(2) $$U_{i}=\frac{1}{4}\sum_{j=1}^{4}v_{j}$$

*v*_*j*_ = 0 if neighbor *j* is smaller than reference species *i*; otherwise, *v*_*j*_ = 1.

Uniform angle index (*W*) characterizes the level of regularity among the four nearest neighbors relative to the reference species. It is determined by the proportion of angles (α) that are smaller than the standard angle *α*_0_ (Figure A1 in Appendix A).

(3) $$W=\frac{1}{4}\sum_{j=1}^{4}w_{j}$$

*W*_*i*_ = 1 if *α*_*j*_ < *α*_0_, otherwise *W*_*i*_ = 0, *α*_0_ = 360°/(*n* + 1).

To evaluate dominance, the crown diameter of each individual species was used. The described methods were implemented using the Crancord software (version 1.4; Pommerening 2002; available at http://crancord.org/), which provides built‐in functions for calculating nearest‐neighbor indices and spatial structure parameters. The analysis was conducted using four nearest neighbors (*n* = 4) for each reference species. To mitigate edge effects in the estimation of *M*_*i*_, *W*_*i*_, and *U*_*i*_, we applied the nearest neighbor edge correction method proposed by Pommerening and Stoyan (2006). All analyses were performed in a Windows 10 environment.

#### 2.4.4 Spatial Pattern Analysis

**FIGURE B1:**
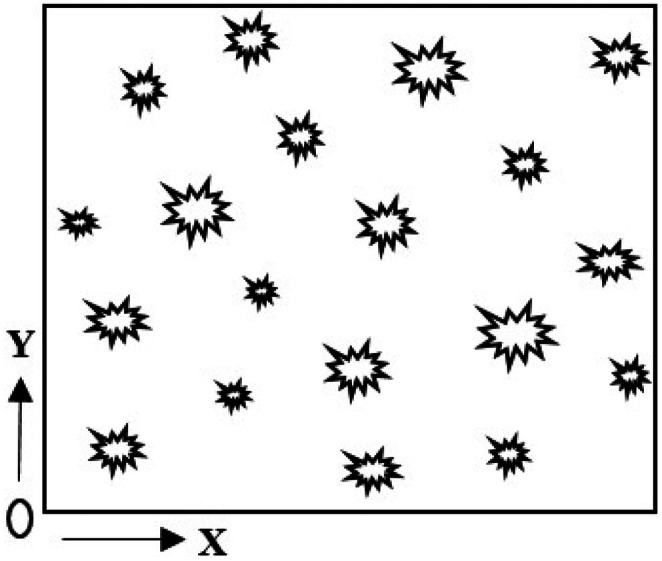
The methods used to record the coordinates of the species inside the plots.

To explore the distribution patterns of alive and dry stands of the target species, point pattern analysis (PPA) was utilized. Scientific names along with X and Y coordinates of all perennial species within the plot were recorded relative to the hypothetical origin of the coordinates in centimeters (Figure B1 in Appendix B) (Ben‐Said 2021).

The main idea of Ripley's K function originates from the number of points within radius *r* around plants, which are divided by the pattern density (*λ*) (Equation 4).

(4) $${\hat{K}}_{\left(r\right)}=A\sum_{i}^{n}\sum_{\neq j}^{n}W_{\mathrm{ij}}I_{r}\left(i,j\right)/n^{2}$$

In which *A* is the area of the studied plot, *n* is the total number of plants, *r* is the distance or radius studied from the plants, and *I*_*r*_ is the indicator function. If the distance of the central plant (*i*) from the neighbor plant (*j*) is smaller than *r*, it will take the value of one, but if the distance of *i* from *j* is greater than *r*, it will be zero. Also, *w*_*ij*_ is the weight function to correct the marginal effect (Illian et al. 2008).

Ripley's K function is applied in two forms: univariate and bivariate. The univariate function is used when all points on the spatial map represent only the position of the points (plant stands) and lack further information. In this analysis, positive K values indicate a clumped pattern, negative values indicate a uniform pattern, and zero indicates a random pattern of the species (Wiegand and Moloney 2004).

On the other hand, the bivariate function is used when the points on the spatial map include additional information beyond spatial location, such as species, size, or biological stage. This function evaluates the relationship between two groups and identifies whether the groups exhibit attraction (*K* > 0), repulsion (*K* < 0), or independence (*K* = 0). Unlike the univariate analysis, the terms “clumped,” “random,” and “uniform” do not directly apply in bivariate analysis because the focus shifts from individual spatial patterns to interactions between groups (Wiegand and Moloney 2004).

The key to success in using Ripley's K function lies in selecting a proper null hypothesis, which can answer biological questions. The simplest null hypothesis that is extensively used in univariate spatial pattern analysis is the complete randomness (Wiegand and Moloney 2004). In bivariate analyses, the null hypothesis of the independence of alive and dry species of *A. verus* from one another was adopted (Wiegand et al. 2007) and was tested by Monte Carlo simulation. If the K function is within the Monte Carlo boundary, the null hypothesis is supported; otherwise, it is rejected. The Monte Carlo range was calculated at the 95% level by 500 times of simulation (Palmino 2005). Subsequently, the data were employed to determine the point pattern of alive and dry stands of the target species using the Point Map Main software (Ben‐Said 2021).

## 3 Results

### 3.1 Plant Communities Across the Three Study Groups

The results presented in Table 2 highlight distinct differences in the structural characteristics and drying status of *A. verus* individuals across the three site groups (G1, G2, and G3) in 100 m^2^ plots. Group 1 (G1) exhibited the highest total number of plant individuals (averaging 212 per plot) and the highest species richness (averaging 13.4 species), with the maximum recorded richness observed in Site 11 (18 species). This group also showed a significant presence of *A. verus* , with an average of 63 individuals per plot, of which the majority (42) were alive and the remaining (20) were dead. Group 2 (G2) displayed lower total plant individual counts (137 per plot) and lower species richness (9 species on average). However, it had a relatively high number of *A. verus* individuals (averaging 50 per plot), approximately half of which were alive. The average number of dead *A. verus* individuals in G2 (25 per plot) was the highest among the groups, reflecting a more pronounced drying trend for this species in these sites. Group 3 (G3) demonstrated a comparable total number of plant individuals to G1 (averaging 215 per plot) and species richness (13.5 species on average). However, it had the lowest average number of *A. verus* individuals (26 per plot), with only 6 being alive and 20 being dead. This group exhibited the most severe drying trend for *A. verus* , as indicated by the highest mortality rate and the dominance of dead individuals across all plots.

**TABLE 2:** Structural characteristics and drying status of *Astragalus verus* individuals across study sites in 100 m^2^ plots. “No. of individuals of all species” represents the total count of all plant individuals recorded in the plot. “No. of species (Richness)” refers to the number of different species identified within the plot. “No. of *A. verus* ” indicates the total count of *A. verus* individuals, while “No. of *A. verus* alive” and “No. of *A. verus* dead” specify the counts of living and dried *A. verus* individuals, respectively.

Site group	Site number	No. of individuals of all species	No. of species (Richness)	No. of *A. verus*	No. of *A. verus* alive	No. of *A. verus* dead
G1	1	72	9	13	7	4
	5	343	13	181	130	51
	6	190	10	62	40	22
	11	284	18	48	31	17
	12	171	17	10	2	8
Average	—	212	13.4	62.8	42	20.4
G2	2	107	7	85	44	41
	3	182	15	40	21	19
	4	121	5	25	11	14
Average	—	136.7	9	50	25.3	24.7
G3	7	169	12	28	3	25
	8	219	14	29	9	20
	9	297	12	24	6	18
	10	177	16	23	6	17
Average	—	215.5	13.5	26	6	20

Table 3 presents the analysis of various species diversity indices across three groups of Sites (G1, G2, and G3), each comprising multiple study plots. While no statistically significant differences were observed between the groups (*p* > 0.05 for all indices), some variations in diversity metrics were evident. Dominance values ranged from 0.12 (indicating highly diverse communities) to 0.64 (indicating strong species dominance). Equitability values varied from 0.41 to 0.82, reflecting differences in the evenness of species distribution. The Berger–Parker index, which highlights the dominance of the most abundant species, ranged from 0.22 to 0.79 across sites. Additionally, the Chao‐1 estimator for species richness varied notably between plots, with estimated species numbers ranging from 6 to 20, indicating heterogeneity in community composition across the study area.

**TABLE 3:** Species diversity indices across study sites in *Astragalus verus* habitats in central Iran.

Site group	G1					G2			G3				*p*
Sites	1	5	6	11	12	2	3	4	7	8	9	10	
Dominance	0.24	0.32	0.25	0.12	0.19	0.64	0.19	0.5	0.22	0.45	0.41	0.25	0.139
Simpson	0.75	0.67	0.74	0.87	0.8	0.35	0.8	0.49	0.77	0.54	0.58	0.74	0.139
Shannon	1.64	1.56	1.53	2.37	2.13	0.8	1.92	0.913	1.77	1.27	1.422	1.88	0.173
Evenness	0.57	0.36	0.46	0.59	0.49	0.32	0.45	0.49	0.49	0.25	0.34	0.41	0.210
Brillouin	1.48	1.5	1.45	2.26	1.98	0.72	1.79	0.85	1.67	1.18	1.35	1.75	0.165
Menhinick	1.06	0.7	0.72	1.06	1.3	0.67	1.11	0.45	0.92	0.94	0.69	1.2	0.501
Margalef	1.87	2.05	1.71	3	3.11	1.28	2.69	0.83	2.14	2.41	1.93	2.89	0.304
Equitability	0.75	0.61	0.66	0.82	0.75	0.41	0.7	0.56	0.71	0.48	0.57	0.67	0.141
Fisher_alpha	2.71	2.67	2.24	4.27	4.69	1.67	3.87	1.05	2.95	3.33	2.5	4.26	0.366
Berger‐Parker	0.34	0.52	0.33	0.22	0.37	0.79	0.32	0.66	0.33	0.65	0.62	0.46	0.160
Chao‐1	10.5	14.5	16	18	20	7	20	6	13	16.5	13.5	17.5	0.371

*Note:* Indices are reported for individual sites because non‐parametric analyses were applied to site‐level values. Group means and dispersion measures were not calculated.

### 3.2 Soil Principal Component Analysis

A principal component analysis (PCA) was performed using all measured soil variables including sand, silt, clay, electrical conductivity (EC), pH, and saturated percentage (SP). PCA summarizes the shared variation among these correlated variables into a smaller number of independent axes. The first PCA axis explained 28.54% of the total variance and the second axis explained 27.40%, giving a cumulative explained variance of 55.94%. Axis 1 represented the main soil gradient and contrasted sites with higher sand content and EC with those characterized by higher SP, silt, and clay. Axis 2 showed a weaker pattern and was primarily associated with variation in pH.

**FIGURE 3:**
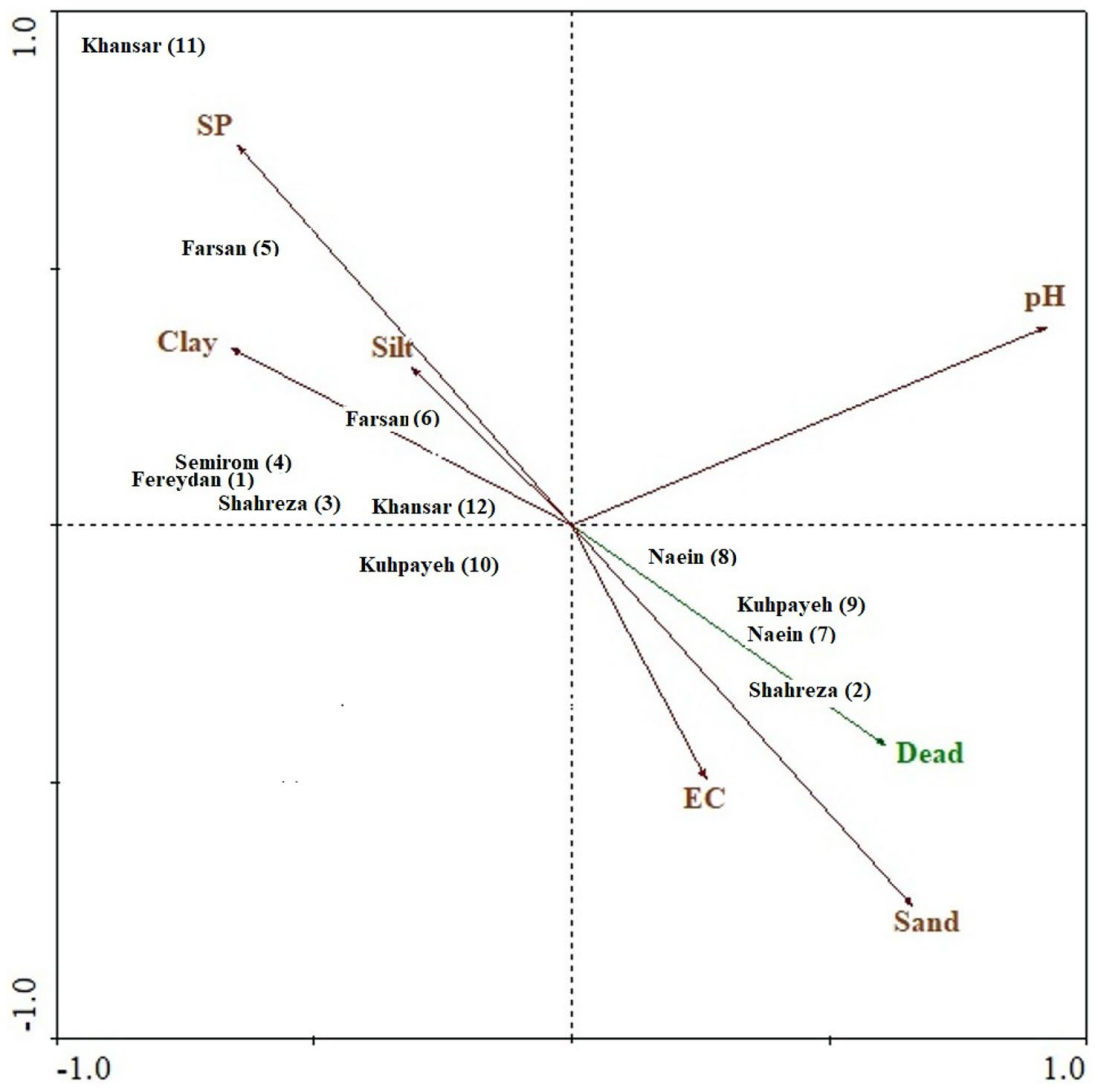
Principal Component Analysis (PCA) triplot showing the relationships among study sites, soil variables (SP = saturated percentage, Clay, Silt, Sand, pH, and EC), and *Astragalus verus* dieback percentage (Dead). Arrows indicate the direction and strength of correlations. Sites are distributed based on their soil characteristics and associated dieback rates.

The PCA ordination (Figure 3) also illustrated clear ecological differentiation among the sites in relation to the dieback percentage of *A. verus* . Sites 7, 8 and 9, which showed the highest dieback values (80.0%, 72.7% and 68.4%), were positioned towards the sand and EC vectors as well as the Dead vector. Site 2, with a moderate dieback rate (48.2%), aligned along the same gradient. In contrast, Sites 1, 3, 4, 5, 6 and 11, which had lower dieback levels, clustered near the SP, silt and clay vectors. These patterns indicate that soil texture and moisture‐related variables are strongly associated with the spatial variation in dieback across the study area.

### 3.3 Nearest Neighbor Analysis

**FIGURE 4:**
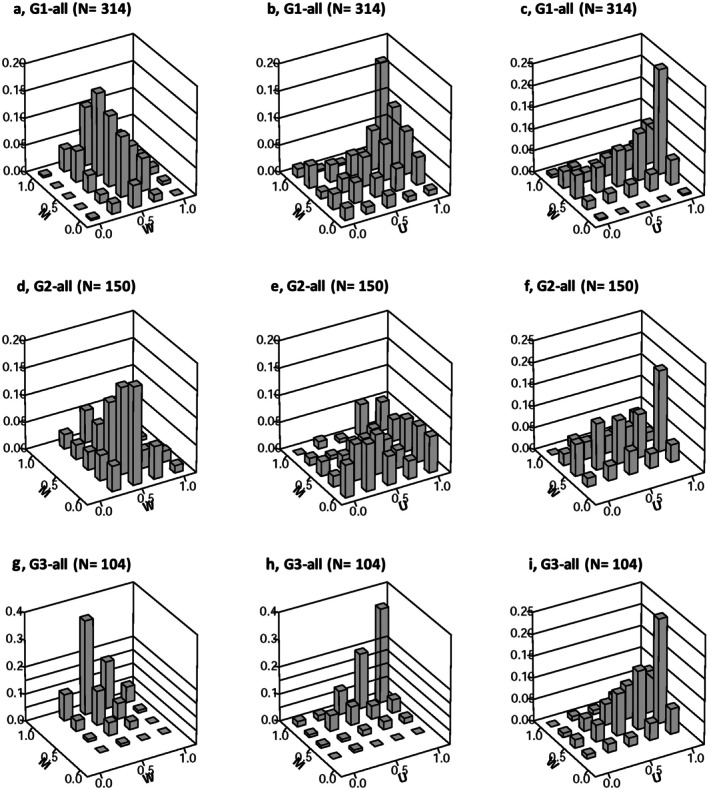
Bivariate frequency distributions of *Astragalus verus* individuals (both alive and dead) across study sites (G1, plots 1, 5, 6, 11, 12; G2, plots 2, 3, 4; G3, plots 7, 8, 9, 10), visualized using nearest neighbor indices. The indices include *M* (Species Mingling Index): Proportion of nearest neighbors differing in species, *W* (Uniform Angle Index): Spatial regularity among neighbors, and *U* (Dominance Index): Proportion of smaller neighbors. Each row corresponds to a site (G1, G2, G3), with subpanels displaying combinations of indices: a, d, g: *M* vs. *W*; b, e, h: *M* vs. *U*; and c, f, i: *W* vs. *U*. The z‐axis indicates frequency, illustrating spatial and size‐related patterns in *A. verus* populations (alive and dead).

The bivariate distribution of *A. verus* species, as represented by M‐W values, demonstrated a consistent trend across the study sites, with the majority of frequency values concentrated between *W* = 0.25 and 0.75 (Figure 4). The peak frequency was consistently observed at *W* = 0.5, with *M* values spanning from 0 to 1.00, suggesting a diverse range of distribution patterns from regular to clumping. Notably, in G3, *M* values skewed towards the higher end, ranging from 0.5 to 1, indicating a greater tendency towards clumping in this specific site. These findings suggest that *A. verus* species primarily display random distribution patterns, albeit with varying levels of interspecies mixing observed across all sites, ranging from minimal to complete mixture. Particularly, G3 exhibited a notably higher degree of interspecies mingling.

**FIGURE 5:**
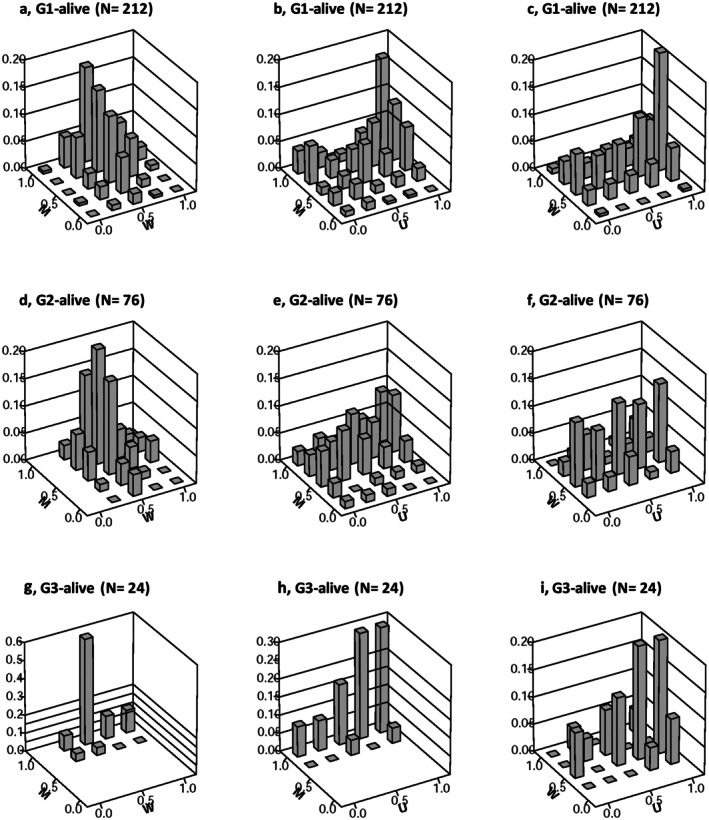
Bivariate distributions of *Astragalus verus* alive individuals across study sites: G1 (plots 1, 5, 6, 11, 12), G2 (plots 2–4), and G3 (plots 7–10). Subpanels display combinations of nearest neighbor indices: a, d, g: *M* vs. *W*; b, e, h: *M* vs. *U*; and c, f, i: *W* vs. *U*. The z‐axis indicates frequency, illustrating spatial and size‐related patterns in *A. verus* alive populations. For index definitions, see Figure 4 or the methods section.

In the case of *A. verus* individuals that are alive, the bivariate *M*‐*W* distributions depict an increase in *M* values from 0 to 1.0, with *W* values spanning from 0.25 to 1.0, notably concentrated at *W* = 0.5 in G1 and G2 (Figure 5a,d,i). Conversely, in G3, *M* values predominantly focused on *M* = 1.0, with *W* ranging from 0.25 to 1.0. Regarding the bivariate patterns of *M*‐*U*, there is an upward trend in M values from 0 to 1.0 and a concurrent increase in *U* values from 0 to 1.0 (Figure 5b,e,j). This indicates that live *A. verus* individuals tend to associate more frequently with neighboring plants of different species (higher mingling) while exhibiting smaller crown sizes relative to their neighbors (lower dominance). Similarly, the *W*‐*U* bivariate patterns exhibit comparable trends across the three study sites, with *W* values ranging from 0.25 to 0.75, prominently concentrated at *W* = 0.5, and *U* increasing from 0 to 1.0 (Figure 5c,f,k). *A. verus* individuals display reduced crown dominance while their distribution ranges from regular to clumped.

**FIGURE 6:**
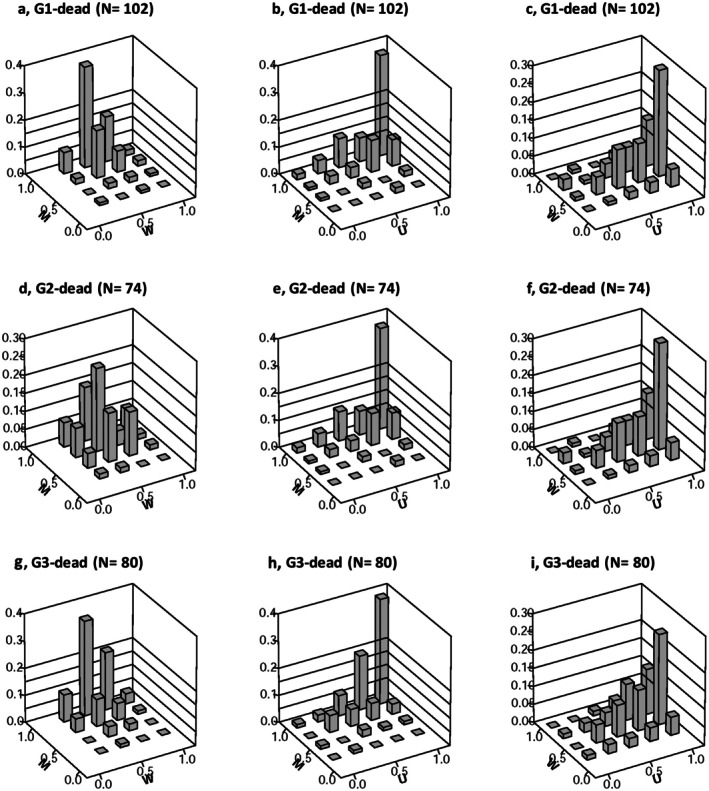
Bivariate distributions of *Astragalus verus* dead individuals across study sites: G1 (plots 1, 5, 6, 11, 12), G2 (plots 2–4), and G3 (plots 7–10). Subpanels display combinations of nearest neighbor indices: a, d, g: *M* vs. *W*; b, e, h: *M* vs. *U*; and c, f, i: *W* vs. *U*. The z‐axis indicates frequency, illustrating spatial and size‐related patterns in *A. verus* dead populations. For index definitions, see Figure 4 or the methods section.

A consistent pattern was observed among *A. verus* dead individuals across all three study sites (Figure 6). The *M*‐*W*‐*U* patterns exhibited a range from *M* = 0.5 to 1.0, *W* = 0.25 to 1.0, and a progressive increase in *U* from 0 to 1.0. These findings indicate that *A. verus* deceased individuals were interspersed among neighboring plants, displaying a distribution ranging from regular to clumped. Additionally, their crown dominance appeared to diminish, suggesting a competitive disadvantage.

### 3.4 Spatial Pattern Analysis

**FIGURE 7:**
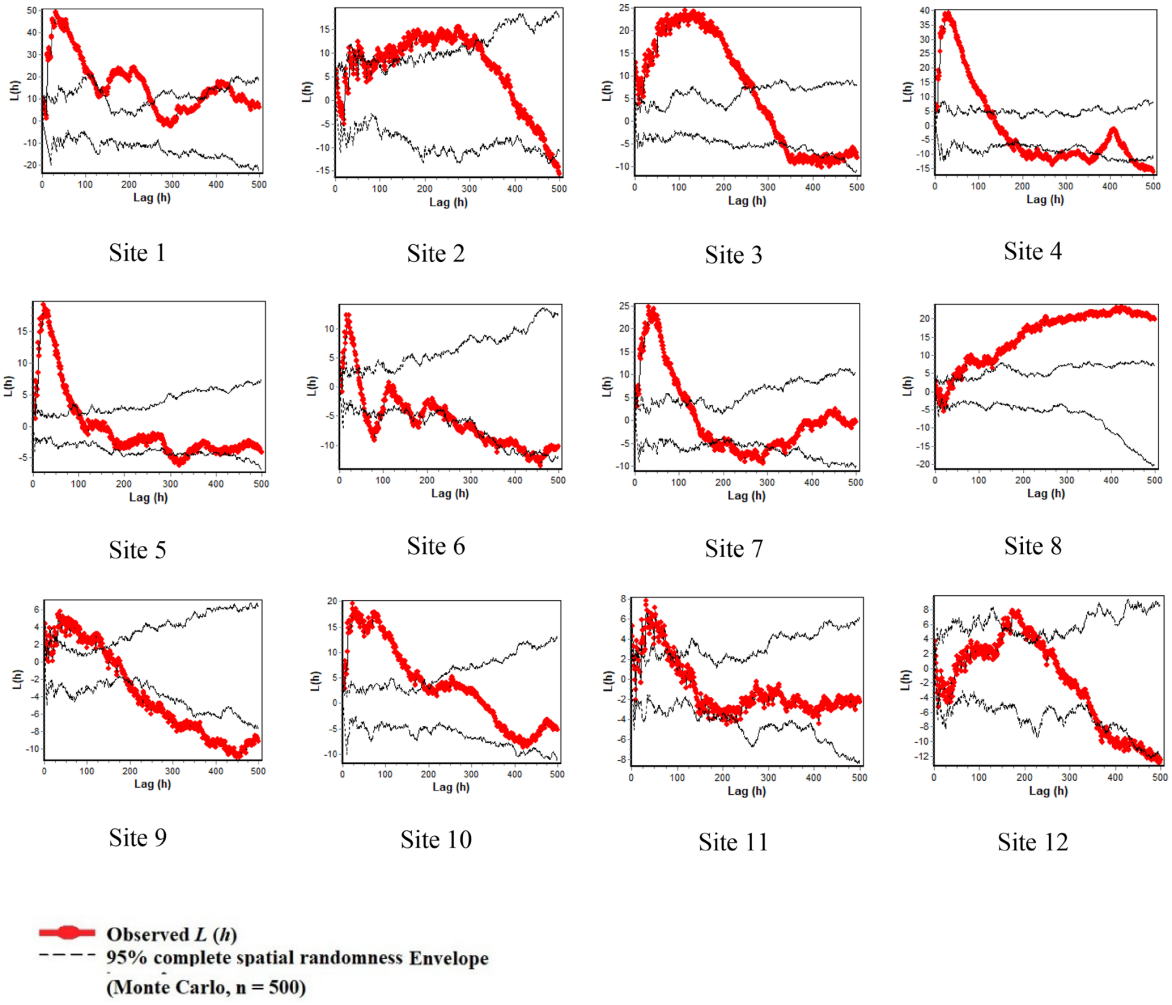
Univariate spatial pattern analysis of all species across 12 rangeland sites in central Iran using Ripley's L (h) function. The red line represents the observed L (h) values for each site, and the black lines show the 95% Monte Carlo simulation envelope generated from 500 random simulations under complete spatial randomness (CSR). Positive deviations of the observed curve outside the upper envelope indicate significant spatial clustering, while negative deviations beyond the lower envelope indicate significant uniformity. Distances (h) are shown in centimeters on the x‐axis, and transformed L (h) values on the y‐axis. Each panel corresponds to one of the 12 study sites.

**FIGURE 8:**
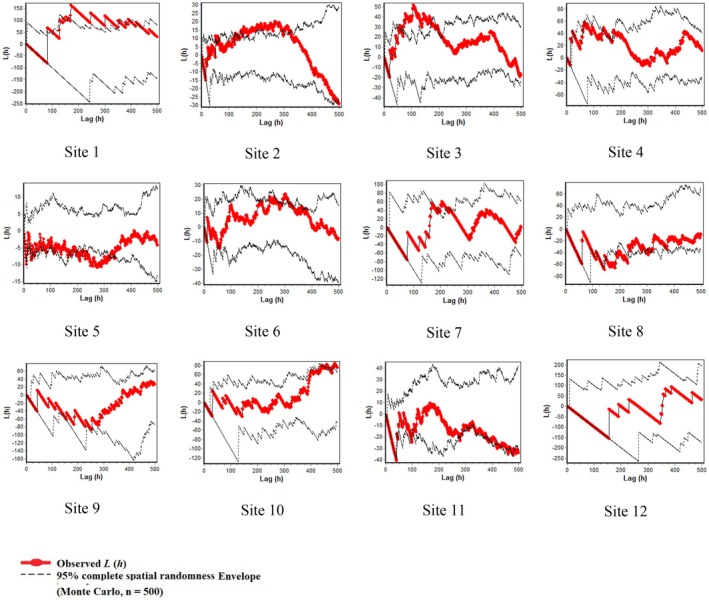
Bivariate spatial pattern analysis of live and dry *Astragalus verus* individuals across 12 rangeland sites in central Iran using Ripley's L_12_ (h) function. The red line represents the observed bivariate L (h) values for each site, and the black lines show the 95% Monte Carlo simulation envelope generated from 500 random simulations under the assumption of independence between the two types (live vs. dry individuals). Positive deviations of the observed curve outside the upper envelope indicate significant spatial attraction between live and dry individuals, while negative deviations beyond the lower envelope indicate significant spatial repulsion. Distances (h) are shown in centimeters on the x‐axis, and transformed L (h) values on the y‐axis. Each panel corresponds to one of the 12 study sites.

**FIGURE C1:**
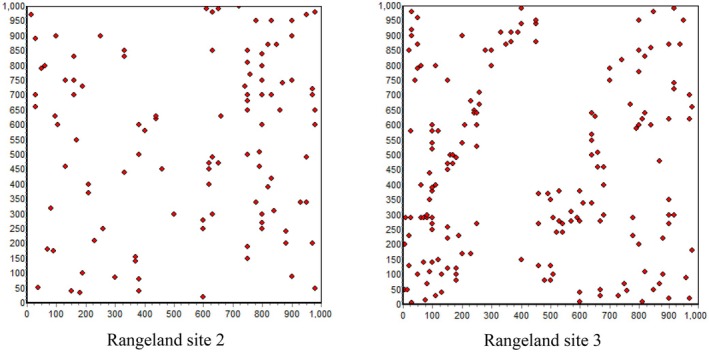
An example of the general schematics of how species are located within plots in the rangeland Sites 2 and 3.

The arrangement of species within plots in rangeland Sites 2 and 3 is illustrated in Figure C1 in Appendix C, providing an overview of their spatial distribution. Figures 7 and 8 present the results of univariate and bivariate analyses, respectively, for all species within each plot, as well as for live and dry *A. verus* plants separately. Our analysis revealed that species distribution within the plots exhibited predominantly clumped patterns at closer distances from the stands, transitioning to more uniform distributions at greater distances (Figure 7). Specifically, the distribution pattern shifted from highly clumped, particularly in proximity to the stands, to increasingly uniform or random patterns as distances from the stands increased across Sites 2, 3, 4, 5, 6, 9, and 12. Conversely, Sites 1, 7, 10, and 11 displayed a transition from severely clumped to random patterns at greater distances. Notably, rangeland site 8 exhibited a consistently clumped distribution pattern across all distance classes. Across all 12 rangeland sites, species distribution within the 0–100 cm distance class exceeded the Monte Carlo boundary, indicative of a clumped pattern, before transitioning to a more uniform pattern below the boundary (Figure 7).

The black lines represent the 95% Monte Carlo simulation envelopes, while the red line indicates the type of species distribution pattern.

Figure 8 illustrates the bivariate pattern analysis depicting the interaction between live and dry stands of *A. verus* . The pattern exhibits a random distribution (within the Monte Carlo boundaries) across 10 sites (from 3 to 12), indicating the independence of live and dry stands from each other. In two sites (Sites 1 and 2), the degree of clumping and attraction between the live and dead *A. verus* stands was minimal (Figure 8).

**FIGURE 9:**
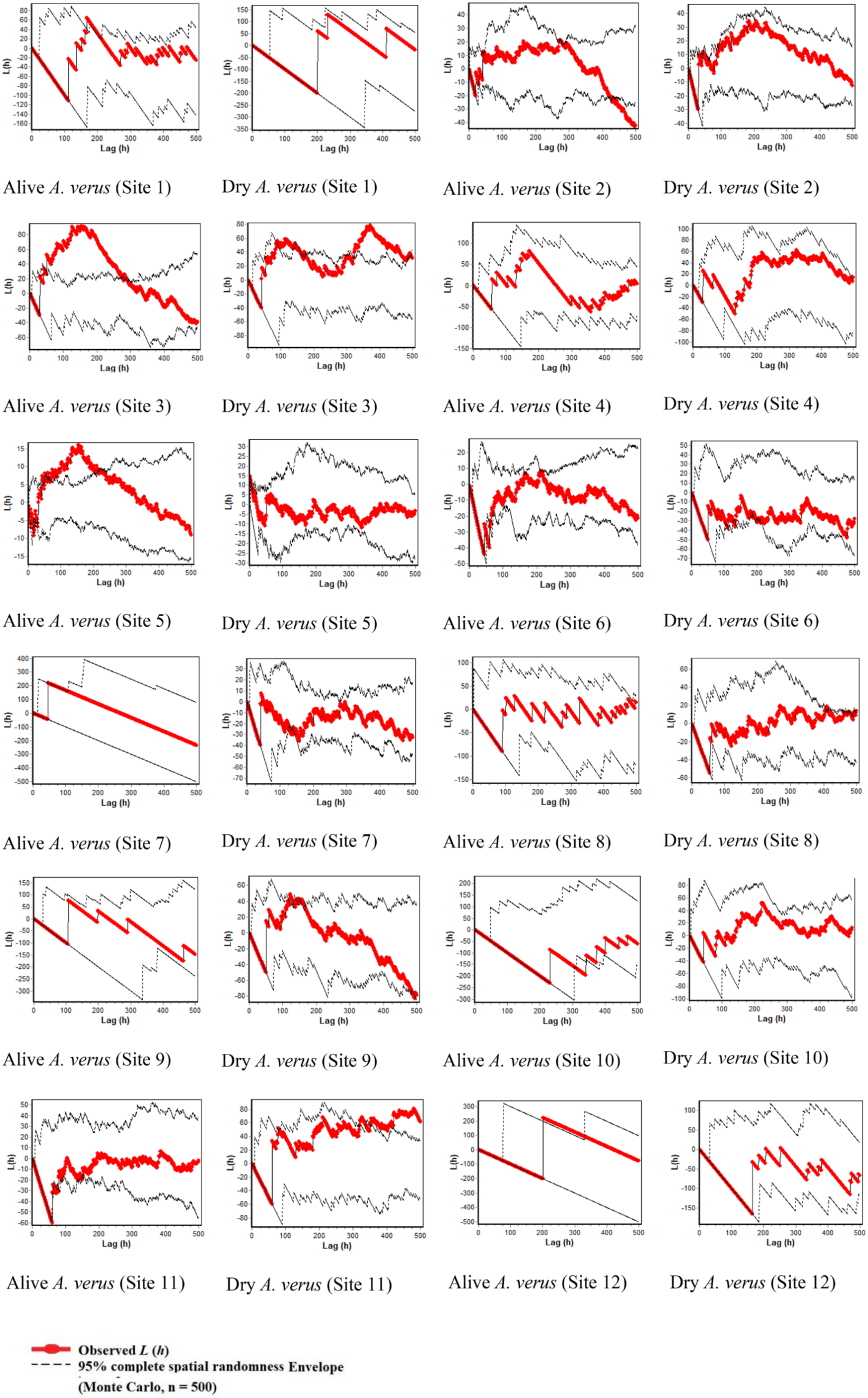
Univariate spatial pattern analysis of all *Astragalus verus* individuals (live and dry combined) across 12 rangeland sites in central Iran using Ripley's L (h) function. The red line represents the observed L (h) values for each site, and the black lines show the 95% Monte Carlo simulation envelope generated from 500 random simulations under complete spatial randomness (CSR). Positive deviations of the observed curve outside the upper envelope indicate significant spatial clustering, while negative deviations beyond the lower envelope indicate significant uniformity. Distances (h) are shown in centimeters on the x‐axis, and transformed L (h) values on the y‐axis.

Figure 9 illustrates the univariate analysis of *A. verus* individuals' plants (both alive and dead). The results indicate that the species exhibits a predominantly random distribution pattern across most rangeland sites. However, clumped distribution is observed only at small distances in Sites 3, 5, and 11, with the distribution falling within the Monte Carlo boundaries for the majority of distances, indicative of a random pattern (Figure 9).

## 4 Discussion

### 4.1 Ecological Drivers of Species Diversity in Semi‐Arid Rangelands

The findings of this study provide valuable insights into the ecological dynamics of *A. verus* and its associated plant communities, advancing our understanding of species interactions, habitat conditions, and the drivers of vegetation patterns in semi‐arid ecosystems. The observed differences in species abundance and composition across the study sites underscore the heterogeneity of ecological conditions within the region. Variations in crown diameter measurements suggest that local environmental factors such as soil characteristics, moisture availability, and topography play critical roles in shaping plant growth and community structure (Clark et al. 2007). These findings highlight the adaptive strategies of *A. verus* in responding to diverse and often harsh environmental conditions, reflecting its resilience and ecological significance in semi‐arid rangelands.

Studies by Bassiri et al. (1989), conducted in grazing exclosures, suggest that perennial grasslands likely represent the climax vegetation of the region under minimal anthropogenic pressures. Their findings indicate that vegetation dominated by perennial grasses can re‐establish itself in the absence of grazing, underscoring the pivotal role of land‐use practices in shaping current rangeland ecosystems. However, decades of overgrazing, land‐use changes, and climatic variability have shifted vegetation dominance towards shrub species like *A. verus* . This transition highlights the ecological resilience of *A. verus* in degraded habitats, while also serving as an indicator of broader ecosystem changes.

The biodiversity indices calculated in this study reveal critical insights into species richness, dominance, and evenness. Subtle shifts in these indices may reflect competitive exclusion processes or adaptive responses by *A. verus* in varying ecological niches. For instance, areas with higher species richness but lower evenness suggest the presence of dominant species, potentially including *A. verus* , that outcompete others in resource‐limited environments. Conversely, regions with balanced dominance and evenness point to more stable and diverse plant communities.

These patterns of diversity are essential for understanding the broader ecological dynamics of arid rangelands. The adaptive strategies of *A. verus* , such as its deep taproot system and drought tolerance, enable it to thrive in these harsh conditions (Masoumi 2006; Sheikhzadeh et al. 2023). Additionally, its ability to coexist with other species, as evidenced by interspecies mingling metrics, highlights its ecological significance in maintaining community structure.

These findings emphasize the importance of integrating species‐specific and community‐level analyses to inform conservation strategies. For example, targeted management efforts could focus on preserving areas with high species richness and monitoring dominant species like *A. verus* to prevent further shifts in community dynamics. Understanding these ecological drivers is critical for developing adaptive management practices that balance conservation and sustainable use of rangeland resources.

### 4.2 Competitive Dynamics and Spatial Interactions of *A. verus*

The nearest neighbor analysis of *A. verus* individuals provided insights into the spatial interactions and competitive dynamics within the studied rangelands. By exploring patterns of mingling (*M*), crown dominance (*U*), and species mixing (*W*), we assessed how *A. verus* interacts with conspecifics and neighboring species, particularly in resource‐limited arid and semi‐arid landscapes.

The peak at *W* = 0.5 suggests a near‐random distribution at the landscape scale, indicative of weak interspecific competition. Alternatively, this pattern could reflect disturbances or mortality events that disrupted previously clumped distributions, highlighting the need for temporal data to distinguish mechanisms. Heightened interspecies mingling in Group 3 sites (G3) indicates that *A. verus* coexists with other species in a more integrated manner, potentially reflecting either localized competition or shared adaptive strategies under environmental constraints. These observations align with broader ecological theories predicting that plant communities in resource‐limited systems balance competitive and facilitative interactions (Wiegand and Moloney 2004).

For living individuals, increased mingling (*M*) and concentration at *W* = 0.5 indicate a tendency to form intraspecific clusters while interacting with neighbors. Such clustering likely enhances survival in harsh environments by maintaining proximity to favorable microhabitats, such as patches with higher soil moisture or nutrient availability. Although increased mingling with reduced crown dominance may suggest competitive suppression, an alternative explanation is that environmental stress limits the ability of individuals to dominate neighbors, emphasizing the importance of considering both biotic and abiotic drivers in NNA interpretations (Getzin et al. 2006; Rayburn 2011).

Spatial patterns of deceased individuals further illuminate mortality processes. Consistent *M* values of 0.5–1.0 combined with declining *U* suggest that even in death, *A. verus* remains spatially interspersed with neighboring species. This pattern likely reflects localized mortality driven primarily by environmental stressors such as drought or pest infestations, rather than purely competitive interactions (Maestre 2006; Luis et al. 2008). Understanding these dynamics is crucial for conservation and targeted management, as it reveals how spatial structure influences population resilience.

### 4.3 Mortality Patterns and Implications for Population Resilience

The spatial distribution patterns of *A. verus* reflect a complex interaction between ecological factors and environmental stressors. In addition to these abiotic influences, biotic interactions, particularly facilitation, may also contribute to the spatial configuration of living individuals. In semi‐arid ecosystems, the stress‐gradient hypothesis predicts that facilitative interactions become increasingly important under harsh conditions, where neighboring plants help buffer drought, temperature extremes, or soil limitations (Callaway and Walker 1997; Brooker et al. 2008). The clustering of living *A. verus* individuals may therefore reflect positive interactions such as microclimate amelioration, enhanced soil moisture retention beneath shrub canopies, or protection from herbivory. Such nurse plant effects have been widely documented in arid and semi‐arid systems and can increase seedling establishment and adult survival (Flores and Jurado 2003; Maestre et al. 2009). Considering these potential facilitative processes provides a more balanced ecological interpretation and suggests that both positive and negative biotic interactions may shape the persistence of *A. verus* under stressful conditions.

At smaller spatial scales, the clumped distribution of *A. verus* aligns with its reproductive and dispersal strategies and indicates the influence of localized environmental factors such as soil moisture retention, topography, and microclimates (Jamali et al. 2020). At larger scales, the transition to random or uniform patterns in living individuals suggests adaptive strategies to reduce competition for limited resources such as water, nutrients, and sunlight (Shi et al. 2024).

In contrast, dead individuals consistently form clumped patterns, highlighting localized dieback events. These clusters may result from environmental stressors including drought, soil properties, and pest infestations such as Xylotrechus ilamensis (Sheikhzadeh et al. 2023), or may reflect historical competition for microsites with favorable conditions. Comparisons of dead clusters with soil texture and moisture data indicate that edaphic factors amplify stress effects; although the relative contributions of biotic versus abiotic mechanisms require further longitudinal or manipulative studies.

The PCA results further support the hypothesis that soil texture and moisture availability are key drivers influencing *A. verus* viability. Finer textured soils with higher moisture holding capacity appear to buffer individuals against environmental stress, reducing dieback, whereas sandy soils with poor water retention and high salinity exacerbate physiological stress and contribute to plant decline (Wang et al. 2025). These findings help explain the clustered mortality patterns observed in specific areas and emphasize the importance of microsite characteristics in determining individual survival.

Such patterns are consistent with observations in other ecosystems. For example, Ma et al. (2024) reported similar mortality clustering in tropical karst forests, where environmental heterogeneity strongly influenced plant persistence. Maestre et al. (2006) observed analogous trends in Mediterranean grasslands, linking clumped mortality to microhabitat variability, while Ludwig et al. (2004) highlighted the joint role of competition and abiotic stress in savanna systems. These comparisons illustrate a broader ecological principle: in water limited ecosystems, plant mortality often follows environmental gradients, emphasizing the importance of site‐specific factors in shaping demographic outcomes.

The consistently clumped pattern observed in certain sites, particularly site 8, further demonstrates how localized environmental factors such as soil properties, microclimates, and human disturbances influence spatial distribution. Ripley's K function revealed mixed spatial patterns, with living plants showing random distributions and dead plants clumped, underscoring the heterogeneity of stress impacts. Integrating these spatial and environmental insights provides critical guidance for targeted conservation and restoration efforts for *A. verus* populations.

A key methodological consideration in interpreting these spatial patterns is the influence of scale and plot resolution on the detection of clumping versus randomness. Both Ripley's K function and nearest neighbor metrics are inherently scale dependent, and their outputs can vary with the spatial extent and sampling resolution used (Wiegand and Moloney 2013; Baddeley et al. 2016). Clustering detected at small spatial grains may reflect short‐distance dispersal, facilitation, or microsite heterogeneity, whereas transitions to random or uniform patterns at broader scales may arise from ecological processes operating over larger extents or from decreased sensitivity of the methods at wide radii (Illian et al. 2008). Because nearest neighbor analysis captures only immediate neighborhood interactions, it is particularly sensitive to fine‐scale spatial structure, while Ripley's K accumulates information across increasing distances, which can reveal or obscure broader patterns depending on plot size and shape (Perry et al. 2006). Our plot dimensions were optimized for detecting local interactions among individuals but may not fully capture population‐level spatial processes that manifest at larger scales. Recognizing these scale effects is essential for interpreting the spatial signatures identified here, and future work incorporating multi‐scale sampling or hierarchical spatial models would help clarify how ecological mechanisms interact with methodological constraints to shape the observed structure of *A. verus* populations.

### 4.4 Integrated Spatial Modeling for Adaptive Management

The spatial analysis conducted in this study provides a powerful decision support tool for adaptive rangeland management. By revealing patterns of species distribution, competition, and mortality, these spatial models offer insights into ecosystem functioning. Such information can directly inform targeted interventions. Specifically, combining Ripley's K function, nearest neighbor metrics, and PCA allows managers to detect early signs of ecological stress and prioritize areas for action (Wiegand and Moloney 2004; Khadka et al. 2024).

The identification of mortality hotspots, where dead *A. verus* individuals were consistently clustered, points to localized environmental stressors such as poor soil structure, high salinity, drought exposure, or pest outbreaks (Maestre et al. 2006; Luis et al. 2008; Wang et al. 2025). These areas should be prioritized for detailed ecological assessments to determine site‐specific drivers of decline. Based on these diagnostics, land managers can implement restoration measures such as soil conditioning, erosion control, and localized pest management.

Spatial modeling also revealed patterns of interspecies mingling and crown dominance that highlight zones of intense species interaction. For instance, sites with high mingling indices and moderate crown dominance may reflect competitive equilibrium, where *A. verus* coexists with other species. These sites may serve as ecological benchmarks for restoration. In contrast, regions with low evenness or declining dominance values may indicate competitive exclusion or ecological imbalance. Such areas require adaptive interventions, including reseeding, grazing adjustment, or rotational resting (Baddeley et al. 2016; Getzin et al. 2006; Rayburn 2011).

The capacity of spatial tools to detect early‐stage vegetation shifts creates opportunities for proactive management. Areas where PCA indicated reduced moisture availability or poor soil texture can be flagged for drought‐mitigation strategies. Examples include planting drought‐tolerant species or constructing water retention structures (Jamali et al. 2020; Shi et al. 2024). These efforts are particularly important in semi‐arid environments where resource scarcity limits plant survival and regeneration.

Engaging local communities in rangeland monitoring and decision‐making can enhance the success of spatially informed management. Collaborative programs with ranchers and indigenous groups facilitate early detection of dieback events and enrich restoration planning through traditional ecological knowledge. Training local stakeholders to interpret spatial indicators, such as clustering of dead individuals or shifts in crown metrics, can foster timely responses and improve restoration outcomes.

Moreover, *A. verus* should be recognized as a potential indicator species for rangeland health. Its tolerance to drought, capacity to stabilize soil, and spatial survival patterns make it a strong candidate for guiding restoration. The clustering of living individuals in resource‐rich microsites suggests potential seed‐source zones or core areas for expansion under managed conditions (Masoumi 2006; Sheikhzadeh et al. 2023).

Finally, integrating spatial modeling with long‐term monitoring of climatic variability, grazing pressure, and pest dynamics will strengthen adaptive management. Spatial analysis should not be viewed as an isolated research method but as a dynamic, evolving tool that connects ecological theory with applied decision‐making. Used in this way, spatial models can improve management precision, reduce restoration costs, and build ecological resilience in vulnerable rangeland systems.

## 5 Conclusion

In conclusion, the spatial distribution patterns observed in *A. verus* populations provide valuable insights into the dynamics of rangeland ecosystems. The predominant clumped pattern observed underscores the influence of various factors on species distribution, including seed dispersal mechanisms, reproductive strategies, and environmental heterogeneity. The transition from a highly clumped pattern to a more random distribution at greater distances from the stands highlights the role of habitat features in shaping plant distributions.

The findings from univariate and bivariate analyses underscore the vulnerability of *A. verus* populations to environmental stressors such as drought and pest infestations, emphasizing the importance of conservation efforts. The application of Ripley's K function offers a comprehensive understanding of spatial dynamics, enhancing our ability to inform conservation strategies and address dieback events effectively.

In summary, understanding spatial patterns in vegetation cover is crucial for biodiversity conservation and management planning in rangeland ecosystems. By addressing human interventions and environmental disturbances, stakeholders can preserve species diversity and maintain ecosystem services, contributing to the long‐term sustainability of these valuable ecosystems.

## Author Contributions

**Asieh Sheikhzadeh:** data curation (lead), formal analysis (equal), investigation (equal), methodology (equal), project administration (equal), software (equal), validation (equal), visualization (equal), writing – review and editing (equal). **Hossein Bashari:** conceptualization (equal), data curation (equal), formal analysis (equal), investigation (equal), methodology (equal), resources (equal), software (equal), supervision (equal), validation (equal), visualization (equal), writing – original draft (equal), writing – review and editing (lead). **Mostafa Tarkesh Esfahani:** conceptualization (equal), formal analysis (equal), investigation (equal), methodology (equal), resources (equal), software (equal), supervision (equal), validation (equal), visualization (equal), writing – review and editing (equal). **Hong Hai Nguyen:** formal analysis (equal), software (equal), writing – review and editing (equal).

## Funding

The authors have nothing to report.

## Conflicts of Interest

The authors declare no conflicts of interest.

## Supporting information


**Data S1:** ece373273‐sup‐0001‐DataS1.zip.

## Data Availability

The data supporting the findings of this study are available as Supporting Information submitted with the manuscript and will be made publicly available upon publication.

## References

[ece373273-bib-0001] Aguirre, O. , G. Hui , K. von Gadow , and J. Jiménez . 2003. “An Analysis of Spatial Forest Structure Using Neighbourhood‐Based Variables.” Forest Ecology and Management 183, no. 1–3: 137–145. 10.1016/S0378-1127(03)00102-6.

[ece373273-bib-0002] Azadrooh, H. , M. Farzam , and M. Mesdaghi . 2020. “Effects of Harvest Intensities on Tragacanth Gum Production and Health of *Astragalus verus* .” Iranian Journal of Applied Ecology 9, no. 1: 1–13. 10.47176/ijae.9.1.4535.

[ece373273-bib-0003] Baddeley, A. , E. Rubak , and R. Turner . 2016. Spatial Point Patterns: Methodology and Applications With R. 1st ed. CRC Press.

[ece373273-bib-0004] Bassiri, M. , A. Jalalian , and M. R. Vahabi . 1989. “Investigating Native Plant Species Habitats in Fereydan Region. Project Report. College of Agriculture, Isfahan University of Technology, Isfahan, Iran.”

[ece373273-bib-0005] Battisti, C. , G. Poeta , and G. Fanelli . 2016. An Introduction to Disturbance Ecology, 13–29. Springer.

[ece373273-bib-0006] Ben‐Said, M. 2021. “Spatial Point‐Pattern Analysis as a Powerful Tool in Identifying Pattern‐Process Relationships in Plant Ecology: An Updated Review.” Ecological Processes 10: 1–23.33425642

[ece373273-bib-0007] Brooker, R. W. , F. T. Maestre , R. M. Callaway , et al. 2008. “Facilitation in Plant Communities: The Past, the Present, and the Future.” Journal of Ecology 96, no. 1: 18–34. http://doi/10.1111/j.1365‐2745.2007.01295.x.

[ece373273-bib-0008] Callaway, R. M. , and L. R. Walker . 1997. “Competition and Facilitation: A Synthetic Approach to Interactions in Plant Communities.” Ecology 78, no. 7: 1958–1965. 10.1890/0012-9658(1997)078[1958:CAFASA]2.0.CO;2.

[ece373273-bib-0009] Carter, M. R. , and E. G. Gregorich . 2006. Soil Sampling and Methods of Analysis. CRC Press.

[ece373273-bib-0010] Chen, Y. , and T. J. Shen . 2024. “Distributional Ecology: Opening New Research Windows by Addressing Aggregation‐Related Puzzles.” Diversity and Distributions 30, no. 2: e13818. 10.1111/ddi.13818.

[ece373273-bib-0011] Clark, J. S. , M. Dietze , S. Chakraborty , et al. 2007. “Resolving the Biodiversity Paradox.” Ecology Letters 10, no. 8: 647–659. 10.1111/j.1461-0248.2007.01041.x. 17594418

[ece373273-bib-0012] de Bello, F. , M. Vandewalle , T. Reitalu , et al. 2013. “Evidence for Scale‐ and Disturbance‐Dependent Trait Assembly Patterns in Dry Semi‐Natural Grasslands.” Journal of Ecology 101, no. 5: 1237–1244.

[ece373273-bib-0013] Demichele, D. , E. Belcore , M. Piras , and C. Camporeale . 2025. “Species‐By‐Species Pattern Analysis of Coastal Dune Vegetation.” Journal of Geophysical Research – Biogeosciences 130, no. 2: e2024JG008419. 10.1029/2024JG008419.

[ece373273-bib-0069] Fensham, R. J. , R. J. Fairfax , and D. P. Ward . 2009. “Drought‐Induced Tree Death in Savanna.” Global Change Biology 15, no. 2: 380–387. 10.1111/j.1365-2486.2008.01718.x.

[ece373273-bib-0014] Flores, J. , and E. Jurado . 2003. “Are Nurse‐Protégé Interactions More Common Among Plants From Arid Environments?” Journal of Vegetation Science 14, no. 6: 911–916. 10.1111/j.1654-1103.2003.tb02225.x.

[ece373273-bib-0016] Getzin, S. , C. Dean , F. He , J. A. Trofymow , K. Wiegand , and T. Wiegand . 2006. “Spatial Patterns and Competition of Tree Species in a Douglas‐Fir Chronosequence on Vancouver Island.” Ecography 29, no. 6: 671–682. 10.1111/j.2006.0906-7590.04675.x.

[ece373273-bib-0017] Hui, G. , X. Zhao , Z. Zhao , and K. von Gadow . 2011. “Evaluating Tree Species Spatial Diversity Based on Neighborhood Relationships.” Forest Science 57, no. 4: 292–300. 10.1093/forestscience/57.4.292.

[ece373273-bib-0018] Illian, J. , A. Penttinen , H. Stoyan , and D. Stoyan . 2008. Statistical Analysis and Modelling of Spatial Point Patterns, 560. John Wiley & Sons, Ltd.

[ece373273-bib-0019] Jamali, H. , A. Ebrahimi , E. Ghesareh Ardestani , and F. Pordel . 2020. “Comparison of Distance‐Based Methods of Density Estimation of *Astragalus verus* Olivier and *Astragalus albispinus* Sirj. & Bornm.(Case Study: Steppe Rangeland of Marjan, Chaharmahal‐Va‐Bakhtiari).” Journal of Range and Watershed Management 72, no. 4: 8–16.

[ece373273-bib-0068] Jangman, R. H. G. , C. J. F. Ter Braak , and O. F. R. Van Tangeren . 1987. Data Analysis in Community and Landscape Ecology, 300. Pudoc Wageningen.

[ece373273-bib-0022] Khadka, S. , H. S. He , and S. Bardhan . 2024. “Investigating the Spatial Pattern of White Oak (*Quercus alba* L.) Mortality Using Ripley's K Function Across the Ten States of the Eastern US.” Forests 15, no. 10: 1809. 10.3390/f15101809.

[ece373273-bib-0023] Khodagholi, M. , and R. Saboohi . 2019. “Delineating Changes in Climatic Variables and Its Impact on the *Astragalus verus* Olivier Habitats in Isfahan Province.” J. Range. Watershed Manage 72, no. 2: 359–374. 10.22059/jrwm.2019.242978.1169.

[ece373273-bib-0024] Lepš, J. 2014. Multivariate Analysis of Ecological Data Using CANOCO 5. 2nd ed. Cambridge University Press.

[ece373273-bib-0025] Li, H. , and J. F. Reynolds . 2023. “Modeling Effects of Spatial Pattern, Drought, and Grazing on Rates of Rangeland Degradation: A Combined Markov and Cellular Automaton Approach.” In Scale in Remote Sensing and GIS, edited by D. Quattrochi and M. Goodchild , 211–230. Routledge.

[ece373273-bib-0028] Ludwig, F. , H. de Kroon , F. Berendse , and H. H. Prins . 2004. “The Influence of Savanna Trees on Nutrient, Water and Light Availability and the Understorey Vegetation.” Plant Ecology 170: 93–105. 10.1023/B:VEGE.0000019023.29636.92.

[ece373273-bib-0029] Luis, M. D. , J. Raventos , T. Wiegand , and C. H. Hidalgo . 2008. “Temporal and Spatial Differentiation in Seedling Emergence May Promote Species Coexistence in Mediterranean Fire‐Prone Ecosystems.” Ecography 31: 620–629. 10.1111/j.0906-7590.2008.05433.x.

[ece373273-bib-0030] Ma, R. , J. Li , Y. Guo , et al. 2024. “Recruitment Dynamics in a Tropical Karst Seasonal Rain Forest: Revealing Complex Processes From Spatial Patterns.” Forest Ecology and Management 553: 121610. 10.1016/j.foreco.2023.121610.

[ece373273-bib-0031] Maestre, F. T. 2006. “Linking the Spatial Patterns of Organisms and Abiotic Factors to Ecosystem Function and Management: Insights From Semi‐Arid Environments.” Web Ecology 6: 75–87. 10.5194/we-6-75-2006.

[ece373273-bib-0032] Maestre, F. T. , R. M. Callaway , F. Valladares , and C. J. Lortie . 2009. “Refining the Stress‐Gradient Hypothesis for Competition and Facilitation in Plant Communities.” Journal of Ecology 97, no. 2: 199–205. 10.1111/j.1365-2745.2008.01476.x.

[ece373273-bib-0033] Maestre, F. T. , J. Cortina , and R. Vallejo . 2006. “Are Ecosystem Composition, Structure, and Functional Status Related to Restoration Success? A Test From Semiarid Mediterranean Steppes.” Restoration Ecology 14, no. 2: 258–266. 10.1111/j.1526-100X.2006.00128.x.

[ece373273-bib-0034] Magurran, A. E. 2021. “Measuring Biological Diversity.” Current Biology 31, no. 19: 1174–1177. 10.1016/j.cub.2021.07.049 34637726

[ece373273-bib-0035] Maltez‐Mouro, S. , L. V. García , T. Marañón , and H. Freitas . 2007. “Recruitment Patterns in a Mediterranean Oak Forest: A Case Study Showing the Importance of the Spatial Component.” Forest Science 53, no. 6: 645–652. 10.1093/forestscience/53.6.645.

[ece373273-bib-0038] Masoumi, A. A. 2006. Iran's Astragalus. 5th ed, 786. Forest and Rangeland Research Institute.

[ece373273-bib-0040] Morera, B. , P. J. Garrote , T. Wiegand , D. Ayllón , and J. M. Fedriani . 2025. “Invariant Spatial Pattern Across Mediterranean Scrublands in the Iberian Pear (*Pyrus bourgaeana*).” Ecology and Evolution 15, no. 1: e70757. 10.1002/ece3.70757. 39839340 PMC11748438

[ece373273-bib-0041] Nizamani, M. M. , J. P. Cubino , A. J. Harris , L. Y. Guo , and H. F. Wang . 2023. “Spatial Patterns and Drivers of Plant Diversity in the Tropical City of Sanya, China.” Urban Forestry & Urban Greening 79: 127818. 10.1016/j.ufug.2022.127818.

[ece373273-bib-0042] Padilla‐Martínez, J. R. , C. Paul , K. Husmann , J. J. Corral‐Rivas , and K. von Gadow . 2024. “Grouping Tree Species to Estimate Basal Area Increment in Temperate Multispecies Forests in Durango, Mexico.” Forest Ecosystems 11: 100158. 10.1016/j.fecs.2023.100158. PMC9610172 36297787

[ece373273-bib-0043] Palmino, R. L. 2005. “Spatial Distribution Patterns of Trees in a Seasonally Dry Forest in the Cerros de Amotape National Park, Northwestern Peru.” Peruana de Biología 12, no. 2: 317–326.

[ece373273-bib-0044] Perry, G. L. W. , N. J. Enright , B. P. Miller , and B. B. Lamont . 2008. “Spatial Patterns in Species‐Rich Sclerophyll Shrublands of Southwestern Australia.” Journal of Vegetation Science 19: 705–716. 10.3170/2008-8-18441.

[ece373273-bib-0045] Perry, G. L. W. , B. P. Miller , and N. J. Enright . 2006. “A Comparison of Methods for the Statistical Analysis of Spatial Point Patterns in Plant Ecology.” Plant Ecology 187, no. 1: 59–82. 10.1007/s11258-006-9133-4.

[ece373273-bib-0046] Podlech, D. , A. A. Maassoumi , and S. H. Zarre . 2012. “Papilionaceae VII: *Astragalus* L. V.” In Flora Iranica, No. 179, edited by K. H. Rechinger , 1–312. Naturhistorisches Museum Wien / Akademische Druck‐ und Verlagsanstalt.

[ece373273-bib-0070] Pommerening, A. 2002. “Approaches to Quantifying Forest Structures.” Forestry 75, no. 3: 305–324. 10.1093/forestry/75.3.30.

[ece373273-bib-0047] Pommerening, A. , and D. Stoyan . 2006. “Edge‐Correction Needs in Estimating Indices of Spatial Forest Structure.” Canadian Journal of Forest Research 36, no. 7: 1723–1739. 10.1139/x06-060.

[ece373273-bib-0048] Pommerening, A. , and D. Stoyan . 2008. “Reconstructing Spatial Tree Points From Nearest Neighbor Summary Statistics Measured in Small Sub‐Windows.” Canadian Journal of Forest Research 38: 1110–1122. 10.1139/X07-222.

[ece373273-bib-0049] Ramón, P. , E. Velázquez , A. Escudero , and M. De la Cruz . 2018. “Environmental Heterogeneity Blurs the Signature of Dispersal Syndromes on Spatial Patterns of Woody Species in a Moist Tropical Forest.” PLoS One 13, no. 2: e0192341. 10.1371/journal.pone.0192341. 29451871 PMC5815593

[ece373273-bib-0050] Rayburn, A. P. 2011. “Causes and Consequences of Plant Spatial Patterns in Natural and Experimental Great Basin (USA) Plant Communities. PhD Dissertation, Utah State University, Logan, UT, 162.”

[ece373273-bib-0051] Ripley, B. D. 1977. “Modeling spatial patterns.” Journal of the Royal Statistical Society, Series B 39, no. 2: 172–212.

[ece373273-bib-0052] Safaei, M. , M. Tarkesh , H. Bashari , and M. Bassiri . 2018. “Modeling Potential Habitat of *Astragalus verus* Olivier for Conservation Decisions: A Comparison of Three Correlative Models.” Flora 242: 61–69. 10.1016/j.flora.2018.03.001.

[ece373273-bib-0053] Scaggs, S. A. , X. Wu , Z. Syed , J. Lebowitz , R. Qin , and S. S. Downey . 2025. “The Landscape Ecology of Swidden: A Global Comparison Indicates Swidden Landscape Mosaics Contribute to Vegetation Diversity at Intermediate Levels of Disturbance. bioRxiv.” 10.1101/2025.03.05.641554.

[ece373273-bib-0054] Sheikhzadeh, A. , M. Tarkesh Esfahani , and H. Bashari . 2023. “Predicting the Occurrence and Decline of *Astragalus verus* Olivier Under Climate Change Scenarios in Central Iran.” Arid Land Research and Management 37, no. 4: 577–601. 10.1080/15324982.2023.2177905.

[ece373273-bib-0055] Shi, Y. , X. Gao , C. Lang , and C. Luo . 2024. “A Method for Determining the Spatial Pattern of Forest Trees Based on the Uniformity Theory.” Journal of Forest Research 35, no. 1: 121. 10.1007/s11676-024-01773-z.

[ece373273-bib-0056] Stamatellos, G. , and G. Panourgias . 2005. “Simulating Spatial Distributions of Forest Trees by Using Data From Fixed Area Plots.” Forestry 78, no. 3: 305–312. 10.1093/forestry/cpi028.

[ece373273-bib-0057] Trifković, S. , and H. Yamamoto . 2008. “Indexing of Spatial Patterns of Trees Using a Mean of Angles.” Journal of Forest Research 13: 117–121. 10.1007/s10310-007-0055-3.

[ece373273-bib-0058] Tsitsoni, T. , D. Karamanolis , G. Stamatellos , and P. Ganatsas . 2003. “Spatial Pattern and Connection of Tree Diameter Classes in *Pinus halepensis* M. Stands After Wildfire. In: Proceedings of the 8th International Conference on Environmental Science and Technology, Lemnos Island, Greece.”

[ece373273-bib-0059] Vahabi, M. R. 2005. “Determining Effective Habitat Indices for the Use of *Astragalus verus* and *Astragalus gossypinus* in Isfahan Province. PhD Dissertation, Tehran University, Iran, 212.”

[ece373273-bib-0060] Von Gadow, K. , and G. Y. Hui . 2002. “Characterizing Forest Spatial Structure and Diversity.” In Sustainable Forestry in Temperate Regions, edited by L. Björk , 20–30. SUFOR, University of Lund.

[ece373273-bib-0061] Wang, R. , Q. Yang , Z. Deng , and W. Nian . 2025. “The Research on Soil‐Plant‐Climate Interactions: An Integrated Assessment of Water Management and Drought Resilience.” Advanced Research in Research 5, no. 1: 456–476. 10.50908/arr.5.1_456.

[ece373273-bib-0062] Wiegand, T. , S. Gunatilleke , N. Gunatilleke , and T. Okuda . 2007. “Analysing the Structure of a Sri Lankan Tree Species With Multiple Scales of Clustering.” Ecology 88, no. 12: 3088–3102. 10.1890/06-1350.1. 18229843

[ece373273-bib-0063] Wiegand, T. , and K. A. Moloney . 2004. “Rings, Circles, and Null Models for Point Pattern Analysis in Ecology.” Oikos 104: 209–229. 10.1111/j.0030-1299.2004.12497.x.

[ece373273-bib-0064] Wiegand, T. , and K. A. Moloney . 2013. Handbook of Spatial Point‐Pattern Analysis in Ecology. CRC press.

[ece373273-bib-0066] Yu, X. , Y. Liu , S. Niu , W. Zhao , C. Fu , and Z. Chen . 2024. “Structure, Functions, and Interactions of Dryland Ecosystems.” In Dryland Social‐Ecological Systems in Changing Environments, 69–107. Springer Nature Singapore.

[ece373273-bib-0067] Zarre, S. , A. A. Maassoumi , and D. Podlech . 2008. “Papilionaceae V: *Astragalus* L. III.” In Flora Iranica, edited by K. H. Rechinger , vol. 177, 1–124. Naturhistorisches Museum Wien.

